# Reducing Hindering Job Demands: The Role of Belief in Life as a Zero-Sum Game and Workload

**DOI:** 10.3390/ijerph181910036

**Published:** 2021-09-24

**Authors:** Marta Roczniewska, Bogdan Wojciszke

**Affiliations:** 1 Faculty in Sopot, SWPS University of Social Sciences and Humanities, 03-815 Warsaw, Poland; bwojcisz@swps.edu.pl; 2 Department of Learning, Informatics, Management, and Ethics, Karolinska Institutet, 171 77 Stockholm, Sweden

**Keywords:** belief in life as a zero-sum game, job crafting, workload, hindering demands, challenging demands, suppression effect

## Abstract

When individuals engage in job crafting by decreasing their job demands, the workload of their teammates rises. Pursuing self-interest at the expense of others requires holding a belief about the antagonistic nature of human relations. The present research demonstrates how belief in life as a zero-sum game (BZSG) shapes workplace behaviors. Our two studies—one cross-sectional and one time-lagged—support our predictions that a strong BZSG weakens proactivity and increases the tendency to decrease one’s job demands at the expense of others. We also observed a suppression effect: workload triggers a reduction in job demands indirectly by activating BZSG, while the direct link between workload and reducing hindering job demands is negative. The results are important for both theory and practice because they delineate the conditions that prompt the avoidance of job demands by the employees.

## 1. Introduction

When faced with heavy workload, employees may choose to proactively lower their work level by reducing job demands that threaten their performance. Decreasing hindering job demands entails performing behaviors that aim to minimize the physical, cognitive, and emotional strains of the job to meet individual needs and abilities [1]. Employees may, for instance, avoid contact with problematic clients, postpone making difficult decisions, or organize their work so that it will not be strenuous. However, job demands that are minimized or avoided by one individual do not just “vanish into the vacuum”. In fact, research has demonstrated that when one member of a team admits to decreasing his or her level of job demands, other team members report an increased workload [2]. Therefore, reducing job demands comes with a consequence: a person is prepared to dump his or her workload on colleagues. Who would be willing to engage in this behavior at the expense of others? In this research, we use the social axiom of a belief in a zero-sum game (BZSG) [3] to explain people’s tendency to engage in decreasing the level of their job demands in the workplace without consideration for their colleagues. BZSG describes a belief about the antagonistic nature of social relations based on the notion that one’s gain comes at the price of another’s loss [3]. We predict, therefore, that cherishing a strong belief in life as a zero-sum game would predispose individuals to reduce their job demands at the expense of colleagues.

The purpose of this paper is twofold. First, we aim to increase understanding of the phenomenon of reducing job demands by explaining why and when people are willing to engage in this behavior. Our focus is on investigating the role of one social axiom, i.e., a belief that life is a zero-sum game. In two studies, one cross-sectional and one time-lagged, we examine how BZSG is linked to decreasing job demands. Another purpose of this research is to disambiguate the role of workload in predicting the reduction of job demands (Study 2). Here, we postulate that an excessive workload activates BZSG, which subsequently leads to a tendency to reduce the demands that one confronts at work. Following the literature on stress appraisal [4], we identify a challenging aspect of workload and expect a direct and negative relationship between workload and reducing job demands. By disambiguating these effects, we further Job Demands–Resources (JD–R) theory [5], demonstrating the circumstances under which job demands are reduced.

## 2. Decreasing Hindrance Job Demands as Job Customization

Some aspects of jobs require physical or psychological effort from employees, for example, excessive noise, time pressure or the emotional demands of the role. These taxing workplace characteristics are called *job demands* [6], and they are associated with certain physiological and psychological costs [5]. Some job demands have a *challenging* nature, i.e., although they are appraised as stressful, they provide the potential for growth and may have a positive effect on an individual [7]. Examples include time pressure or the cognitive demands of the job. Other types of job demands, like organizational constraints or interpersonal conflicts, are a *hindrance* to effective goal pursuit and therefore influence an individual negatively [7]. Intense demands can trigger the health impairment process because they exhaust an individual [8], but to the rescue come *job resources*: those aspects of the environment that buffer the negative aspects of job demands [9]. The presence of resources is linked with higher work engagement [10]. In line with JD–R theory [8,9,11], it is vital for employees to find a balance between their job demands and job resources to perform well and to be satisfied with their jobs.

When job demands exceed employee capabilities, individuals may proactively decrease the levels of strain to stop the health impairment process and support job performance [12]. This bottom-up action is one strategy within a job customization repertoire that has been labeled *job crafting* [1]. Job crafting aims to change the characteristics of one’s job to meet personal needs and preferences [13]. However, lowering one’s job demands is not the only type of behavior employees may engage in to craft their jobs; in fact, it tends to be the rarest one [14]; or at least, the one that is reported most reluctantly. Based on the JD–R model, Tims and Bakker [1] proposed that job crafting consists of three dimensions: (a) increasing structural and social job resources; (b) increasing challenging job demands; and finally (c) decreasing hindering job demands. Hence, when employees experience a misfit between their preferences and job demands or the resources available in the workplace, they may seek more resources to help them deal with the demands (e.g., ask colleagues for advice or seek development opportunities), or they may seek more challenges when their job is not stimulating enough (e.g., take on new projects [1]. These behaviors have been labeled *expansion-oriented crafting* [15]. Finally, when employees are overwhelmed with hindering job demands, they may try to reduce them, e.g., minimize contact with clients or colleagues who cause problems. Decreasing one’s job demands, although it may at first seem to fix the problem, has been linked with multiple negative consequences, like lower job satisfaction and work engagement, higher turnover intention, and less extra-role performance [14]. Given these destructive outcomes, it is important to investigate when and why people are willing to engage in decreasing their job demands.

Research on the job-crafting phenomenon, which has expanded over recent years [16], pointed to several antecedents to decreasing hindering job demands that relate to both to the work environment and to the individuals. A meta-analysis performed by Rudolph and colleagues [14] demonstrated a weak and negative link between autonomy and the tendency to reduce job demands: higher levels of job independence seem to prevent employees from decreasing hindering job demands. Autonomy provides a sense of control that facilitates redesign and allows employees to tackle demands in a more constructive way. However, some authors have found that managers who are less experienced in their jobs use their job autonomy to reduce their job demands [17]. This finding indicates that the reduction of job demands by managers with short tenure may be triggered by their lower ability to deal with the high demands of their role; therefore, it may elucidate how demands cause reduction-oriented crafting when they surpass an individual’s resources and when external factors (i.e., high job autonomy) allow for it.

On the employee end, an avoidance temperament [18], prevention focus [19], neuroticism [14] and dark triad traits [20] have been positively linked with a tendency to engage in decreasing job demands. These results display, in addition to the environmental factors, the importance of personality traits in predicting how employees will react to hindering job demands. However, individual orientations and beliefs may also play a significant role in job-redesign behaviors. To the best of our knowledge, no previous research has addressed the role that beliefs may play in shaping the job-crafting actions that reduce hindering job demands. Studies show that self-efficacy is positively related to job crafting in the form of increasing resources and challenges; however, it is not linked with decreasing job demands [14,21]. Optimism, as a tendency to make positive attributions about one’s success and future events, has been demonstrated to predict expansion-oriented crafting; again, however, it is not related to reduction-oriented crafting [22].

Given the above, we deem that there is scarcity of knowledge about how beliefs and convictions may predispose individuals to reduce the hindering demands they face at their jobs. However, we argue that general beliefs, labeled as *axiomatic*, are especially relevant here. Axiomatic beliefs are often a very powerful driver of behavior: presumed to be true and formed as an outcome of personal and culturally shared experiences transferred through socialization, axiomatic beliefs are often applied without being questioned or realized [23]. It seems, therefore, important to understand their role in shaping organizational behaviors. Here, we propose that reducing job demands may be the aftermath of a generalized expectation that life is a zero-sum game.

## 3. Belief in a Zero-Sum Game (BZSG)

BZSG is a broad-spectrum belief about the nature of social relations, where one’s interests opposes those of others [3]. It is based on the following assumption: there is a finite amount of goods and resources in the world; hence, one person’s win automatically turns another person into a loser (and vice versa). BZSG may lead to a more callous pattern of functioning, where people want to prevent a negative scenario for themselves and therefore act selfishly. Studies have demonstrated that BZSG affects cognition (e.g., elicits distrust; [3], emotions (e.g., sadness and anxiety; [24]), and behaviors (e.g., lack of cooperation; [25] or and a tendency to engage in a negative, tit-for-tat reciprocity [24]. Interestingly, BZSG varies not only among individuals but also among nations [3]. For example, countries with a lower gross domestic product (GDP) have a higher aggregate score on the BZSG scale: belief in a zero-sum game seems to arise in countries where resources are scarce [3].

BZSG is a social axiom [26] and thus different from an individual belief [3]. Namely, the latter is typically specific and applies to a particular (often narrow) range of situations. In contrast, social axioms may be viewed as highly abstract “generalized expectancies” that relate to social behavior across a variety of contexts and targets [3], including the work environment. Research in organizational settings showed a negative relationship between BZSG and work input. Individuals who believe that life is a zero-sum game are less willing to use their abilities to the fullest when performing their jobs, they are less keen on improving their actions, and they don’t believe that the effort they make at work is worth it [27].

The research summarized above gives grounds for a prediction that individuals who are relatively strong in their belief that life is a zero-sum game will be more willing to decrease their job demands. BZSG prevents them from worrying whether the increased strain will affect their colleagues because these individuals anticipate that their colleagues would do the same, in line with the negative reciprocity rule. Job demands have been linked repeatedly with employee strain, i.e., when job demands are heavy, employees’ mental and physical resources become drained, which leads to health problems [8]. In the zero-sum game scenario, self-interest is the priority: the employee protects him- or herself without considering the well-being of colleagues or organizational success. Therefore, the best strategy is to prevent the health impairment process by reducing the sources of stress—minimizing or avoiding job demands that cause strain.

**Hypothesis** **1.** *There Is a Positive Link between BZSG and Decreasing Hindering Job Demands*.

We also predict that BZSG impairs employee proactivity. First, employees high in BZSG may not be willing to ask for help or advice because they distrust others and may fear that their lack of competence will be used against them. Thus, their distrust and belief in negative reciprocity means that they are less likely to seek social job resources. Moreover, BZSG does not promote extra effort because it may be costly: it is more important to preserve one’s resources than to spend them [28]. Additionally, BZSG is linked with sadness and anxiety [3], and these negative emotions relate to behavioral withdrawal [29]. Therefore, we expect that individuals who share a belief that life is a zero-sum game will be neither interested in increasing their autonomy and learning opportunities at work (increasing structural job resources) nor inclined to take on extra work (increasing challenging job demands). Overall, we expect that

**Hypothesis** **2.** *There Is a Negative Link between BZSG and Expansion-Oriented Job Crafting, i.e., Seeking Job Resources and Challenges*.

### 3.1. Study 1

#### 3.1.1. Method

Procedure and participants. All data were collected via ll data collected via online forms implemented into Qualtrics. Respondents were Polish employees recruited with network sampling by nine student research assistants. We followed Demerouti and Rispens’s guidelines [30] on assuring the quality of students-recruited samples. Each student was instructed to first obtain informed consent from potential participants and then to send the survey link. Participant recruitment criteria were as follows: (a) working for an organization in the public or private sector (not self-employed or freelance), (b) employed for at least 6 months in the current workplace, and (c) working at least 20 h a week (part-time employment equivalent to half of full-time employment in Poland). Two hundred thirteen participants agreed to take part in the study; however, 46 did not fully complete the survey, which resulted in a final sample of 167 individuals with no missing data. Of these participants, 72% were women. On average, participants were 34 years old (*SD* = 7.50) and had worked in the current workplace for 3 years.

Measures. *Belief in a Zero-Sum Game* was measured using a 12-item BZSG scale [3]. Unlike Adamska and colleagues [27], we did not focus participants’ attention on their beliefs regarding their current workplace but were interested in a generalized belief as a social axiom. Hence, participants were asked to rate their general agreement with a set of statements (e.g., “Life is so devised that when somebody gains, others have to lose.”, “Life is like a tennis game—a person wins only when others lose.”) using a scale from 1 (strongly disagree) to 7 (strongly agree). The Cronbach’s alpha for this scale was *α* = 82.

*Job crafting* was assessed using the Polish adaptation [31] of the job-crafting scale [32]. This instrument allows us to measure 4 types of job-crafting behaviors: increasing structural job resources (e.g., “I try to develop myself professionally”; *α* = 0.78), increasing social job resources (e.g., “I ask others for feedback on my job performance”; *α* = 0.74), increasing challenging job demands (e.g., “When an interesting project comes along, I offer myself proactively as a project coworker”; *α* = 0.85), and decreasing hindering job demands (e.g., “I try to ensure that my work is emotionally less intense”; *α* = 0.75). The respondents indicated how often they had engaged in each of the behaviors (1 = never, 5 = very often) over the last month.

#### 3.1.2. Results and Discussion

The datasets generated and analyzed during the current study are available in the Open Science Framework repository, https://goo.gl/8N25af (accessed on 29 July 2021). Table 1 presents the descriptive statistics and intercorrelations of the variables of Study 1. In line with Hypothesis 1, we observed a weak but positive relationship between BZSG and decreasing hindering job demands (*r* = 0.23, *p* = 0.003). Employees who scored higher on the BZSG scale were more likely to reduce their job demands. As predicted by Hypothesis 2, the relationships between BZSG and expansion-oriented job-crafting behaviors (i.e., increasing structural and social job demands, as well as increasing job challenges) were negative.

In this study, we were interested in finding how social axioms, specifically BZSG, affect employee behavior in the workplace. We expected and found that individuals who are inclined to believe that life is a zero-sum game reduce their job demands more often than individuals who score low on the BZSG scale. BZSG is a social axiom and, as such, is unlike the constructs that have been previously studied as predictors of job crafting. However, BZSG shares qualities with traits that have previously been linked with reducing job demands: a tendency towards anxiety and sadness (neuroticism [14]), the violation of social rules and callousness (Machiavellianism [20]), a sense of entitlement (narcissism [20]), and withdrawal (cynicism [32]). In this sense, the results we observed here replicate previous findings but also bring a novel spectrum of factors that—apart from personality and work environment—explain why employees engage in decreasing their job demands. Here, we underline the importance of axiomatic beliefs in determining why some people are more willing than others to react to their job demands by reducing them.

Moreover, we observed that BZSG inhibits proactivity in the workplace. First, a higher BZSG is linked with a lower chance of seeking social job resources. BZSG leads to a negative evaluation of social relations in an organization; previous research has demonstrated that a negative workplace climate hinders knowledge sharing [33]. Hence, by neglecting social relations as a source of support and seeing others as possible opponents, BZSG prevents employees from asking colleagues or supervisors for help. Next, BZSG is also negatively linked to increasing the use of structural job resources: BZSG employees are less likely to seek learning opportunities, develop their competences, or use their capabilities to the fullest. This could result from the sadness and anxiety that are linked with BZSG. Previous research has demonstrated that negative emotions do not promote positive psychological functioning: individuals are less likely to cultivate their strengths and seek developmental opportunities when they experience negative emotions [34]. This may explain why BZSG inhibits the increase of structural job resources among employees. We also observed that BZSG prevents individuals from seeking challenging job demands: they tend to take on new tasks or seek stimulating workplace activities less frequently. This pattern of results replicates previous findings demonstrating a negative link between BZSG and work input [27].

### 3.2. Study 2

Overall, Study 1 showed that a conviction that social life, including that in the workplace, is a zero-sum game hinders initiative, knowledge sharing, work contribution and extra-role behaviors. Given this unfortunate pattern of workplace behaviors, it seems important to examine the factors that can trigger the process in which high BZSG individuals decrease job demands. Certain states (e.g., lack of resources, [3]) or contextual cues (e.g., rivalry in the workplace) may activate beliefs that life is a like a zero-sum game, and further affect human cognitions, emotions, and behaviors. In line with these assumptions, previous research demonstrated a mediating role of zero-sum beliefs between poor sleep and lower life satisfaction [35]. When people perceive prevalent societal normlessness. In a series of studies, Sirola and Pitesa [36] demonstrated that worse economic periods are associated with a more zero-sum construal of success and make people less likely to help others. We argue that heavy workload may be a contextual cue to activate zero-sum construal. The potentially threatening aspect of the workload activates BZSG as a protection strategy: if work life is a zero-sum game, one must avoid losses and act to prevent negative outcomes for oneself. Differences in coping emerge most clearly when individuals are placed in stressful situations: demanding conditions reveal with particular clarity individuals’ characteristics, including their strategies for coping [37]. Moreover, ample research has demonstrated that job demands lead to other undesirable organizational acts: counterproductive work behaviors (CWB). Shoji and colleagues [38] observed a positive link between quantitative workload and workplace deviance among police officers. In another study, it was found that employees were more likely to make derogatory remarks or be condescending towards clients when they faced excessive job demands [39].

It seems only logical that people are more inclined to reduce their job demands under heavy workloads. The demands of high volume and fast-paced work can exhaust the mental and physical resources of an employee [38,39]. The strain may deplete energy and lead to health problems [5]. Surprisingly, a meta-analysis [14] did not confirm a link between a heavy workload and decreasing job demands. We believe this may be because the workload activates two simultaneous processes that cancel each other out. First, in line with the logic explicated above, we predict that a heavy workload cues zero-sum beliefs, which further facilitate the decision to reduce one’s job demands. The relationship between a heavy workload and reducing hindering job demands is thus indirect. We postulate the following hypotheses:

**Hypothesis** **3.** *A Greater Workload Is Linked with Higher BZSG*.

**Hypothesis** **4.** *BZSG Mediates the Relationship between Workload and Decreasing Hindering Job Demands*.

However, a second mechanism is the direct link between workload and reducing job demands. Challenging job demands differ from hindering job demands in that, although they are also appraised as stressful, they have the potential to support employees’ goals [40]. Workload can be stimulating; it shows employees that they can attain more difficult goals and learn from the experience. Challenging job demands motivate employees to develop their knowledge and skills [7]; they offer mastery experiences that help build self-efficacy [41]. Workload may therefore become a significant way to boost one’s competence and job satisfaction. Supporting this argument, the previously-mentioned meta-analysis [14] found a positive link between workload and increased challenging demands: a greater volume and higher pace of work predicted the search for more job demands of a challenging nature, e.g., adding complexity to the tasks performed at work. Overall, we expect that individuals may be less willing to reduce their demands when they experience a heavy workload once BZSG is out of the picture. It is because workload can be stimulating, promote development of knowledge and, which can boost self-efficacy.

**Hypothesis** **5.** *The Direct Relationship between Workload and Decreasing Hindering Job Demands Is Negative*.

#### 3.2.1. Method

Procedure and participants. We conducted a time-lagged cross-sectional study. Participants were recruited via the SONA research management system panel among respondents who were tagged in the database as employed (but not self-employed). They were all first-year psychology students. Participation was anonymous and voluntary, and participants earned required course credit for participation. Ninety two individuals participated in the study at Time 1, and one hundred and seven participated at Time 2, which was placed on the platform approximately 1 month after Time 1. Eighty four employees (74 women) completed the scales at both measurement times and formed the final study sample. On average, participants were 30 years old and had worked for their current employer for 5 years. Seventy percent of them worked 40 h a week or less, while others reported working more.

#### 3.2.2. Measures

We measured workload and BZSG at Time 1, and we measured job crafting at Time 2. We used the same instrument used in Study 1 to measure BZSG (*α* = 0.83) and reducing hindering job demands (*α* = 0.71). We applied the JC Scale in full, but due to space limitations, we only describe the results of the subscale ‘decreasing hindering job demands’, which is the focus of our paper.

To measure *workload,* we used the Quantitative Workload Inventory [42] adapted to Polish [43]. This measure consists of five items that allow us to assess respondents’ perceptions of their work in terms of its volume and pace. The items refer to the quantity of tasks, the effort required to perform them, and the time allotted for task completion (e.g., “How often does your job leave you with little time to get things done?”; “How often do you have to do more work than you can do well?”). The Cronbach’s alpha for scale reliability in the current study was *α* = 0.79.

#### 3.2.3. Results and Discussion

##### Analysis Strategy

The datasets generated and analyzed during the current study are available in the Open Science Framework repository, https://goo.gl/8N25af (accessed on 29 July 2021). We conducted a simple mediation analysis (Model 4, using SPSS 24 with the PROCESS macro [44], applying 10,000 bootstrapping repetitions with bias-corrected confidence intervals to estimate the indirect effect [45]. Workload at T1 acted as a predictor, BZSG measured at T1 acted as a mediator, and reducing hindering demands at T2 acted as a dependent variable.

##### Hypothesis Testing

In line with Hypothesis 3, workload was positively related to BZSG (Path a), estimate = 0.26, SE = 0.14, LLCI = −0.01, ULCI = 0.54. However, the confidence intervals included 0 and this effect should be treated with caution. Replicating the pattern of results observed in Study 1 and as predicted by Hypothesis 1, higher BZGS predicted the more frequent reduction of hindering job demands (Path b), estimate = 0.21, SE = 0.09, LLCI = 0.02, ULCI = 0.39. The total effect of workload on reducing hindering job demands was not significant (Path c), estimate = −0.19, SE = 0.12, LLCI = −0.43, ULCI = 0.05. Table 2 demonstrates the total, direct, and indirect effects of workload on reducing hindering job demands. Interestingly, the estimate of the direct effect was negative, while the estimate of the indirect effect through BZSG was positive, representing an inconsistent mediation [46]. Yet, the indirect effect included 0, and therefore it should be regarded with caution (Because the LL CI value was close to 0, we performed several mediation analyses in PROCESS macro with bootstrapping (10,000 bootstrap samples) to check the robustness of the findings. In most cases, LL CI were slightly below 0 (c. −0.0001), while in others they excluded 0. We additionally performed the analysis in JASP in the same setup but using Maximum Likelikood as an estimator. Here, the value of LL CI was c. 0.0003, supporting the existence of the indirect effect. However, with other estimators the effect was not robust.).

The fact that the links between workload and reducing hindering job demands may be both positive (direct) and negative (indirect), points to the fact that workload may simultaneously trigger two conflicting processes. First, more workload activates BZSG, which then predicts reducing job demands. This pattern is consistent with Hypothesis 4; however, because the indirect effect included 0, it should be treated with caution. Simultaneously, there is a direct negative relationship between workload and reducing job demands, i.e., individuals who face heavy workloads are less motivated to reduce their job demands. This is in line with Hypothesis 5.

Overall, in Study 2 we found mixed support for our predictions. We successfully replicated the pattern observed in Study 1: BZSG was again positively linked to decreasing hindering job demands. As predicted, a stronger belief in life as a zero-sum game enhanced the probability of employees engaging in reducing their job demands. Here, this connection was found between variables tested across two separate time points (a one month lag), which adds credibility to the results obtained in Study 1 and lowers the probability that common method bias affected our results [47].

In line with our predictions, workload was positively related to BZSG. In high-demand situations, such as an elevated amount and speed of work, when the self-regulatory system is taxed, an individual’s personality traits, coping strategies, and core beliefs will have a more profound impact on shaping behaviors [20,37]. A greater workload seemed to activate the axiomatic belief that life is a zero-sum game. However, the confidence intervals of this effect included 0 and, therefore, Hypothesis 3 could not be fully supported.

Interestingly, we also detected that BZSG acts as a suppressor variable to the negative relationship between workload and reducing job demands. Thus, the results indicate that, for employees without BZSG, a higher workload is linked to the less frequent reduction of hindering job demands by employees. Workload is a challenging job demand: although it does positively relate to exhaustion [40], it also predicts higher job satisfaction, stronger organizational commitment [48], and better job performance [7]. Therefore, employees who experience a greater workload may be less willing to decrease their job demands because they thrive under its challenging aspects.

## 4. General Discussion

### 4.1. Theoretical Contributions

In this research, we sought to investigate how the belief that life is a zero-sum game shapes workplace attitudes and behaviors. Across two studies, we demonstrated that this social axiom makes people more likely to reduce hindering job demands. Individuals high in BZSG minimize the strains associated with their job more often than those who are low in BZSG. Employees manifest this tendency by evading interactions with difficult clients or emotionally demanding colleagues, minimizing the cognitive or physical strain related to the work, or deferring making decisions. Individuals who believe that life is a zero-sum game reduce hindering job demands regardless of the fact that this behavior increases their colleagues workload [2] and can negatively affect achieving company aims [49]. The willingness to decrease hindering job demands despite the interpersonal or organizational consequences suggests a certain mentality: these employees accept that others must lose when they gain; any other approach would put their own interests at stake. This research furthers knowledge on the antecedents of decreasing job demands, which has received relatively less attention than expansion-oriented crafting strategies.

In Study 1, we also observed that BZSG stifles proactivity. Employees who believe that life is a zero-sum game do not capitalize on their resources by seeking new opportunities or by increasing their workplace challenges. They are less likely to invest effort into developing their capabilities or expanding their knowledge and competences to increase their fit to the job. These individuals do not seek advice or feedback from their colleagues, possibly due to their negative view of social relations and a fear that incompetence might put them at disadvantage. These findings are consistent with those showing that BZSG is linked with loneliness [50], as well as distrust at an individual level and societal cynicism at a country level [3]. This undesirable pattern of behaviors not only has negative outcomes for the organizations, which are now, more than ever, looking for proactivity within the workforce [51], but also for the employee, as an unaddressed misfit between one’s preferences and the job one performs has been repeatedly linked with negative consequences (for a meta-analysis see: [52]. When individuals are not able to perform their jobs in line with their strategic inclinations, they stand a higher chance of experiencing exhaustion and disengagement [53]. Hence, BZSG puts employees at risk of developing job burnout. Along with the other negative correlates of BZSG previously detected—like a negative vision of the social world, the delegitimization of the social system, and low life satisfaction—maladaptive job-crafting patterns put individuals who score high in BZSG in a “losing” position [3].

The present research expands the outcomes of BZSG to the workplace domain. Importantly, in these two studies, participants’ focus was on their general beliefs rather than their specific workplace situation with regard to the nature of resources and social relations. Hence, this research points to the importance of individuals’ core beliefs in shaping counterproductive workplace behaviors (CWB). This result overlaps with research demonstrating the importance of personality traits in predicting CWB [54] or decreasing job demands [18,19,20]. Our findings open a new avenue of research on the predictors and antecedents of BZSG in the workplace. However, while personality traits have biological foundations and are shaped by the environment, e.g., upbringing or culture, BZSG is also shaped by the economic context of the country (i.e., GDP). This suggests the existence of sociological predecessors to reducing job demands among employees, which have not received much attention in the past.

Study 2 allowed us to demonstrate that BZSG can be prompted by heavy workload. Specifically, those participants who reported an increased volume and pace of work also expressed a stronger belief that life is a zero-sum game. Simultaneously, we observed that, when controlling for BZSG, workload was negatively related to reducing job demands. The existence of two conflicting processes that are activated by workload provides an explanation as to why the meta-analysis by Rudolph and colleagues [14] found the relationship between workload and decreasing hindering job demands to be close to zero. One mechanism captures the taxing nature of job demands, which increases the impact of core personal characteristics, like personality traits or axiomatic beliefs, on attitudes and behaviors. By this logic, exhaustion allows for some of these characteristics to emerge and influence actions. This might be especially true for undesirable malevolent characteristics that are usually “under control” because of their social undesirability [55]. The other mechanism points to a potentially growth-triggering component of many job demands such as time pressure or workload. Employees acquire new abilities when they deal with these challenges, which may overall have a positive impact on their competences. Furthermore, learning that one is able to perform certain acts despite hitting barriers boosts self-efficacy [56], which increases the probability of approaching (rather than avoiding) challenges in the future [57]. Our finding allows for a better understanding of boundary conditions that explain when workload is linked with employee avoidance and when it is linked to seeking even greater job demands.

### 4.2. Limitations and Future Research

Below, we recognize and address certain limitations of this research. First, in both studies, we collected data based on self-reports. This type of data collection relies heavily on respondents’ willingness to admit to their attitudes and behaviors. Both BZSG and decreasing hindrance job demands are perceived as socially undesirable; hence, individuals may not be eager to disclose them. It is therefore notable that, although the items measuring these two constructs may be difficult to endorse, we were still able to observe meaningful relationships between BZSG and decreasing hindering job demands. One way to avoid self-reported single-source declarative data would be to ask colleagues or supervisors about their ratings of respondents’ attitudes or behaviors. However, BZSG and decreasing job demands may be hard to observe [1], and people may be reluctant to disclose their colleague’s negative beliefs or attitudes, fearing it may get those coworkers into trouble.

Furthermore, we cannot draw definite conclusions about the causal relationships between the variables we tested because Study 1 and part of Study 2 were conducted using a cross-sectional design, which may introduce common method variance (CMV) [47]. We used different response categories and timeframes (BZSG concerns ‘general’ beliefs, while job crafting was measured as experienced over the previous month) to reduce the risk of CMV as an explanation for the relationships observed in our studies [47]. In Study 2, we also replicated the positive link between BZSG and reducing hindering job demands using a time-lag design, separating these two variables by a 1-month interval. This gives more credibility to this result, although future researchers should consider an experimental study design to test causal links between BZSG and reducing job demands. Moreover, while we predicted and observed that a higher workload is linked with a stronger BZSG, this effect was weak, and the confidence intervals included 0. Thus, this link should be treated with caution and replicated. It also seems plausible that BZSG increases the probability of perceiving that one has a greater workload (reversed causality). Individuals who believe that life is a zero-sum game feel like they are losing, and they may attribute their low performance to having a greater workload (or job demands in general). Hence, BZSG serves as a justification mechanism explaining why they wound up where they are. This is a strategy to protect one’s self-esteem in the event of failure and resembles self-handicapping: in anticipation of a failing performance, individuals create or identify more obstacles to attribute external blame [58]. More objective workload data (e.g., nurse-to-patient ratio, teaching hours, or tasks completed) are needed to better understand the causality between workload and BZSG. Another suggestion could be laboratory or field experiment where workload is manipulated to observe whether excessive work instantiates a win-or-lose mindset. In particular, it may be interesting to test these relations in teamwork settings to inspect the consequences in the amount of work among the teammates.

A limitation to the generalizability of our findings relates to the fact that the sample in Study 2 was obtained from one university’s participant pool, where most students pursue psychology. As a result, the majority of the sample consisted of women (88%), who were relatively young, and did not have a long work experience. It could also be argued that psychology students might have different coping skills compared to the general population, e.g., engage in less dysfunctional behaviors or seek social support to a higher extent. To generalize our findings, future research should investigate the relationships in question among individuals with different educational experience, diverse in age and tenure, as well as balanced in terms of gender.

Previous research revealed differences between nations with regard to the levels of BZSG, explaining these variances based on country-level GDP, collectivism and societal cynicism [3]. The fact that these scores aggregate meaningfully on a level higher than the individual level points to the possibility that BZSG can also be a part of the workplace climate. Future studies could therefore investigate predictors of BZSG at the organizational level. For instance, managers’ behaviors and leadership style could elicit a conviction that resources in the workplace, such as developmental opportunities, are scarce. This would increase competition and make subordinates prone to thinking that their interests conflict with the interests of their colleagues. Similarly, organizational culture can also be responsible for the rise of BZSG. The presence of values such as work centrality [59], where competition between employees is not perceived as harmful and opportunities for helping others are not important, may create a workplace where BZSG is prevalent. These higher-level factors that relate to the organization and have the potential to explain BZSG at an individual level may represent a new avenue for research on BZSG.

The fact that the levels of BZSG differ between nations gives grounds for a prediction that cultural differences may also hold for decreasing hindrance job demands. In countries where societal cynicism is relatively high, e.g., Vietnam or Ukraine [3], reducing one’s job demands may be a more common self-regulatory mechanism for achieving a balance between job resources and job demands than in other countries. To the best of our knowledge, these kinds of cross-cultural comparisons have not yet been conducted for the job-crafting phenomenon, and we encourage future research on this topic.

## 5. Conclusions

In this article, we aimed to address the gap in the literature on the influence of axiomatic beliefs on employee behaviors in the workplace. Specifically, we analyzed the extent to which a belief that life is a zero-sum game makes individuals more likely to reduce their job demands. Across two studies, we predicted and found that BZSG explains a reduction of hindering job demands at work and stifles proactivity. Moreover, we disentangled the role of workload in this process, demonstrating that it works both as cue to BZSG that activates coping mechanisms and as a challenging job demand. These findings add to the growing body of research on the antecedents of job crafting in the workplace and expand the range of factors that affect these proactive and reduction-oriented behaviors.

## Figures and Tables

**Table 1:** Descriptive Statistics and Simple Correlations between Belief in a Zero-Sum Game (BZSG) and Four Types of Job-Crafting Behaviors.

	*M (SD)*	1	2	3	4
BZSG (1)	3.06 (0.86)	—			
Job crafting					
Increasing Structural Job Resources (2)	4.27 (0.60)	−0.29 ***	—		
Increasing Social Job Resources (3)	2.84 0.82)	−0.25 **	0.23 **	—	
Increasing Challenging Job Demands (4)	3.59 (0.88)	−0.36 ***	0.59 ***	0.39 ***	—
Decreasing Hindering Job Demands (5)	2.79 (0.76)	0.23 **	−0.07	0.08	−0.17 *

Note. *N* = 167; *** *p* < 0.001, ** *p* < 0.01, * *p* < 0.05.

**Table 2:** The Total, Direct, and Indirect Effect of BZSG in the Relation between Workload and Reducing Hindering Job Demands.

			95% CI	
Effect	Estimate	SE	LL	UL
Total (c)	−0.192	0.119	−0.429	0.045
Direct (c’)	−0.246	0.119	−0.483	−0.010
Indirect	0.054	0.042	−0.000	0.170

Note. *N* = 84. SE = Standard Error, CI = Confidence Interval, LL = Lower Limit, UL = Upper Limit.
